# Pomegranate peel extract alters the microbiome in mice and dysbiosis caused by *Citrobacter rodentium* infection

**DOI:** 10.1002/fsn3.1106

**Published:** 2019-07-07

**Authors:** Nadja S. George, Lumei Cheung, Devanand L. Luthria, Monica Santin, Harry D. Dawson, Arvind A. Bhagwat, Allen D. Smith

**Affiliations:** ^1^ Environmental Microbial and Food Safety Lab Beltsville Agricultural Research Center Agricultural Research Service, Department of Agriculture Beltsville Maryland; ^2^ Diet Genomics and Immunology Lab Beltsville Human Nutrition Research Center Agricultural Research Service, Department of Agriculture Beltsville Maryland; ^3^ Composition Methods Development Lab Beltsville Human Nutrition Research Center Agricultural Research Service, Department of Agriculture Beltsville Maryland; ^4^ Present address: Central Chinmaya Mission Trust Powai Mumbai India

**Keywords:** 16S rRNA, *Citrobacter rodentium*, infectious colitis, microbiome, pomegranate peel extract

## Abstract

Treatment of mice with a pomegranate peel extract (PPX) decreased the pathogenicity of *Citrobacter rodentium* (*Cr*) infections. Here, we investigate the effects of PPX on the microbiome of uninfected or *Cr*‐infected C3H/HeNCr mice by 16S rRNA gene sequencing. Mice were treated with water or PPX for 14 days, feces were collected, and then, the mice were infected with *Cr* and feces collected again at day 6 postinfection. DNA was isolated from the fecal samples and subjected to 16S rRNA gene sequencing to determine the microbial composition. Differences in the composition of the microbiome were observed for untreated and PPX‐treated mice with PPX mice having decreased diversity. PPX treatment decreased the Firmicutes/Bacteroidetes ratio by increasing Bacteroidetes and decreasing Firmicutes levels. The decrease in Firmicutes was driven by a large reduction in *Lactobacillus*. PPX treatment increased the abundance of Proteobacteria and Verrucomicrobiae and decreased Actinobacteria. The relative abundance of *Cr* reached 22% in water‐treated but only 5% in PPX‐treated infected mice. These results suggest that consumption of pomegranate polyphenols altered the microbiome, making it more resistant to displacement by infection with *Cr*, indicating that pomegranate polyphenols may mitigate the pathogenic effects of food‐borne bacterial pathogens.

## 1 INTRODUCTION

Fruits are rich sources of polyphenols and antioxidants. Extracts of a variety of fruits have been tested for their ability to counteract health conditions including obesity, diabetes, and colitis. Grape and apple polyphenols improved the outcome of dextran sulfate sodium (DSS)‐induced colitis in rats (Boussenna et al., 2016; Denis et al., 2016). Fruit extracts, rich in polyphenols, including pomegranate, stimulated the growth of *Akkermansia municiphila* (Anhe, Roy, Pilon, & Dudonne, 2015; Henning, Summanen, Lee, & Yang, 2017; Li, Henning, et al., 2015; Zhang et al., 2018) which is thought to have beneficial health effects (Ottman, Geerlings, Aalvink, Vos, & Belzer, 2017). An increase in *Escherichia coli* (*E. coli*)*,* enterobacteria, and total aerobic counts was found in feces from DSS‐treated rats that decreased in response to treatment with pomegranate extract, or its metabolite urolithin A (Larrosa et al., 2010). Analysis of data from 16S rRNA gene sequencing showed a significant increase in the family *Ruminococcaceae* in feces from DSS‐treated rats receiving pomegranate extract compared with DSS‐treated rats receiving water (Kim, Banerjee, Sirven, & Minamoto, 2017).

For centuries, the pomegranate fruit has been used for its medicinal properties and is widely marketed for its purported health benefits (Shaygannia, Bahmani, Zamanzad, & Rafieian‐Kopaei, 2016). The pomegranate fruit peel is a rich source of punicalins, punicalagins, and ellagic acids, but is an agricultural waste product (Akhtar, Ismail, Fraternale, & Sestili, 2015). Pomegranate peel extract and their principle components punicalagin, ellagic acid, and their metabolites, including urolithin A, have been reported to have antidiabetic, hypolipidemic, antibacterial, and antiparasitic actions (Al‐Mathal & Alsalem, 2012; Finegold, Summanen, Corbett, & Downes, 2014; Howell & D'Souza, 2013; John, Bhagwat, & Luthria, 2017; Mahadwar, Chauhan, Bhagavathy, & Murphy, 2015; Salwe, Sachdev, Bahurupi, & Kumarappan, 2015). Furthermore, it has been reported to reduce the severity of DSS‐ and trinitrobenzene sulfonic acid‐induced colitis (Kim, Banerjee, Ivanov, & Pfent, 2016; Larrosa et al., 2010; Rosillo et al., 2012).

*Citrobacter rodentium* (*Cr*), an *E. coli*‐like bacterium that naturally infects mice, is a robust model to study bacterial pathogenesis and health benefits of food additives and their impact on the microbiota because it replicates many aspects of enteropathogenic bacterial infections in humans, such as the clinically important gastrointestinal pathogens enteropathogenic *E. coli* or enterohemorrhagic *E. coli* (Collins, Keeney, et al., 2014). Infection with *Cr* produces attaching and effacing lesions in the colon, epithelial cell proliferation, an uneven apical enterocyte surface, crypt dilation and hyperplasia, increased cellularity, and mucosal thickening (Collins, Keeney, et al., 2014; MacDonald, Frankel, Dougan, Goncalves, & Simmons, 2003). *Cr* growth is restricted to the colon with little bacterial translocation to systemic compartments, except in highly susceptible mouse strains (Vallance, Deng, Jacobson, & Finlay, 2003). Several lines of evidence suggest that the microbiome plays a significant role in determining the severity of *Cr* infection. Fecal transplant experiments demonstrated that highly susceptible mice can be rendered less susceptible by transplantation of the microbiome from resistant mice (Ghosh et al., 2011; Willing, Vacharaksa, Croxen, Thanachayanont, & Finlay, 2011). Administration of a *Lactobacilli*‐enriched culture to mice reduced *Cr* disease severity (Vong, Pinnell, Maattanen, & Yeung, 2015) while dysbiosis induced by feeding a fiber‐free diet exacerbated disease activity (Desai et al., 2016). Thus, the microbiome plays a key role in determining *Cr* disease severity.

It was observed that pomegranate peel extract (PPX) treatment reduced colon pathology and mortality caused by *Cr* infection of the susceptible mouse strain C3H/HeNCr while not affecting pathogen load, clearance, or immune response (Smith *et al*. manuscript submitted). Since pomegranate consumption has been reported to alter the microbiome, we undertook a study to assess whether PPX treatment altered the microbiome in *Cr*‐susceptible mice, thus contributing to the decreased *Cr*‐induced pathology in PPX‐treated mice.

## 2 EXPERIMENTAL SECTION

### 2.1 Animals

C3H/HeNCr mice were obtained from Charles River Laboratories. Mice were maintained on autoclaved Harland Teklad 7012 Rodent Chow, deionized water, and housed four‐five per cage. Mice were on a 12‐hr light/dark cycle. All experiments were approved by the USDA/ARS Beltsville Institutional Animal Care and Use Committee under protocol number 14–010. Experiments were conducted in compliance with US NRC's Guide for the Care and Use of Animals, the US PHS Policy on Humane Care and Use of Laboratory animals, and the Guide for the Care and Use of Laboratory Animals.

### 2.2 Pomegranate peel extract (PPX) preparation and treatment of mice

Pomegranate peel extracts were prepared and the composition determined by HPLC (John et al., 2017) as described previously (Smith *et al*., submitted). In experiment 1, two groups of five mice were given PPX by oral gavage, either 0.2 ml twice a day (morning and afternoon) or 0.3 ml once a day on weekends. To serve as controls, two groups of five mice were given water by oral gavage. Mice were treated for 2 weeks, and then, fecal pellets were collected to determine the microbiome composition using 16S rRNA gene sequencing.

In experiment 2, two groups of mice (*n* = 4–5) were treated for 14 days with water or PPX and fecal pellets collected to examine microbiome composition, and then, the mice were infected with *Cr.* For the infection, a nalidixic acid‐resistant mutant of *Cr* strain DBS100 (ATCC 51459) was used. A frozen stock of *Cr* was streaked out on an LB agar plate and used to inoculate LB broth that was grown overnight at 37°C with shaking. The following morning, the culture was expanded and grown to an OD_600_ of approximately 1.5. The culture was harvested by centrifugation and resuspended in LB broth. After fasting for 4–6 hr, mice were infected by oral gavage with 0.2 ml (5.0‐10 × 10^9 ^cfu) of the bacterial suspension. The dose was confirmed by retrospective plating. At day six postinfection, when *Cr* colonization is at peak levels, fecal pellets were collected to study the microbiome composition. At day 7 postinfection, fecal pellets were collected, homogenized, and serial dilutions plated on LB/nalidixic acid agar plates to determine the level of *Cr* fecal excretion.

### 2.3 DNA isolation and quantification

Fecal pellets were collected and snap frozen in liquid N_2_. Fecal samples ranging from 80 to 150 mg were homogenized using a Precellys®24 Homogenizer (Bertin Corp., Rockville, MD, USA) and DNA isolated using a ZR Fecal DNA Isolation Kit™ (Zymo Research, Irvine, CA, USA) following the manufacturer's recommendations. DNA samples were further cleaned with the ZR Genomic DNA Clean & Concentrator™‐10 (Zymo Research, Irvine, CA, USA) before quantifying with the Quant‐iT™ dsDNA Assay Kit (Invitrogen™, Carlsbad, CA, USA) using a SpectraMax® M5 Multimode Microplate Reader (Molecular Devices, San Jose, CA, USA). The DNA quality (260/280 ratio of >1.8) was assessed by using NanoDrop 2000^®^ spectrophotometer (Thermo Fisher Scientific, MA, USA) before storage at −20°C.

### 2.4 16S rRNA amplicon sequencing library preparation

16S rRNA amplicon libraries, with the V3‐V4 hypervariable regions of 16S rRNA, were prepared by following the Illumina protocol for the MiSeq System (Experiment 1) (https://support.illumina.com/documents/documentation/chemistry_documentation/16s/16s-metagenomic-library-prep-guide-15044223-b.pdf) or were sent to the University of Maryland Institute for Bioscience and Biotechnology Research DNA Sequencing laboratory for processing and sequencing on a HiSeq 2500 rapid run using the PE300 protocol (Experiment 2). For in‐house library preparation, the quality and size of the libraries were verified by using the Agilent DNA 1000 Kit and run on the Agilent 2100 Bioanalyzer (Agilent Technologies, Santa Clara, CA, USA). The NEBNext® Library Quant Kit for Illumina® (New England Biolabs, Ipswich, MA, USA) was used to quantify each library by using the Mx3005P QPCR System (Agilent Technologies, Santa Clara, CA, USA). Libraries were normalized with 10 mM Tris pH 8.5 to 4 nM before pooling equal volumes. The final library concentration was 6 p.m. with PhiX control v3 (15%, v/v) (Illumina, San Diego, CA, USA). Libraries were sequenced using an Illumina MiSeq sequencer and a 600‐cycle, MiSeq® Reagent Kit v3.

### 2.5 16S reads and data analysis

The demultiplexed sequencing reads from the Illumina MiSeq or HiSeq sequencers were imported into the CLC Genomics Workbench 11.0.1 (CLC Bio, Aarhus, Denmark) and processed accordingly. The quality trimming parameters were set to remove sequences below Phred Score 20, ambiguous nucleotides (maximal 2 nucleotides allowed), and sequences below 100 nucleotides.

The parameters for the open‐reference‐based operational taxonomic unit (OTU) clustering were set to default. Therefore, unique reads were mapped to the Greengene database (Greengenes v13_5; 97% similarity) with the addition to create de novo OTUs. The obtained OTU table for each experiment was filtered to remove all low abundance OTUs (<10). Before the estimation of alpha and beta diversity, the OTUs were aligned via MUSCLE v3.8.31 (Edgar, 2004) to generate a maximum‐likelihood phylogenetic tree (neighbor‐joining tree based on the Jukes–Cantor distances). Alpha diversity was measured using phylogenic index diversity, Chao‐1 bias‐corrected index, and Shannon diversity, whereas Bray–Curtis dissimilarity plot and weighted UniFrac were chosen for the beta diversity. Euclidean heatmaps were generated using CLC Genomics with complete linkage with a fixed number of features set to 200 and minimum count of 10. Additional CLC Genomics statistical analysis included permutational multivariate analysis of variance (PERMANOVA). The OTU table obtained from CLC Genomics was imported into JMP Genomics. The count data were TSS normalized and square root transformed (Hellinger transformation), and analyzed for significance using the Basic Expression Workflow with default settings and a FDR of 0.05. Additional plots and statistics were generated using SigmaPlot 11.0.

## 3 RESULTS

### 3.1 Effect of PPX treatment on the microbiome of uninfected mice

**Figure 1:**
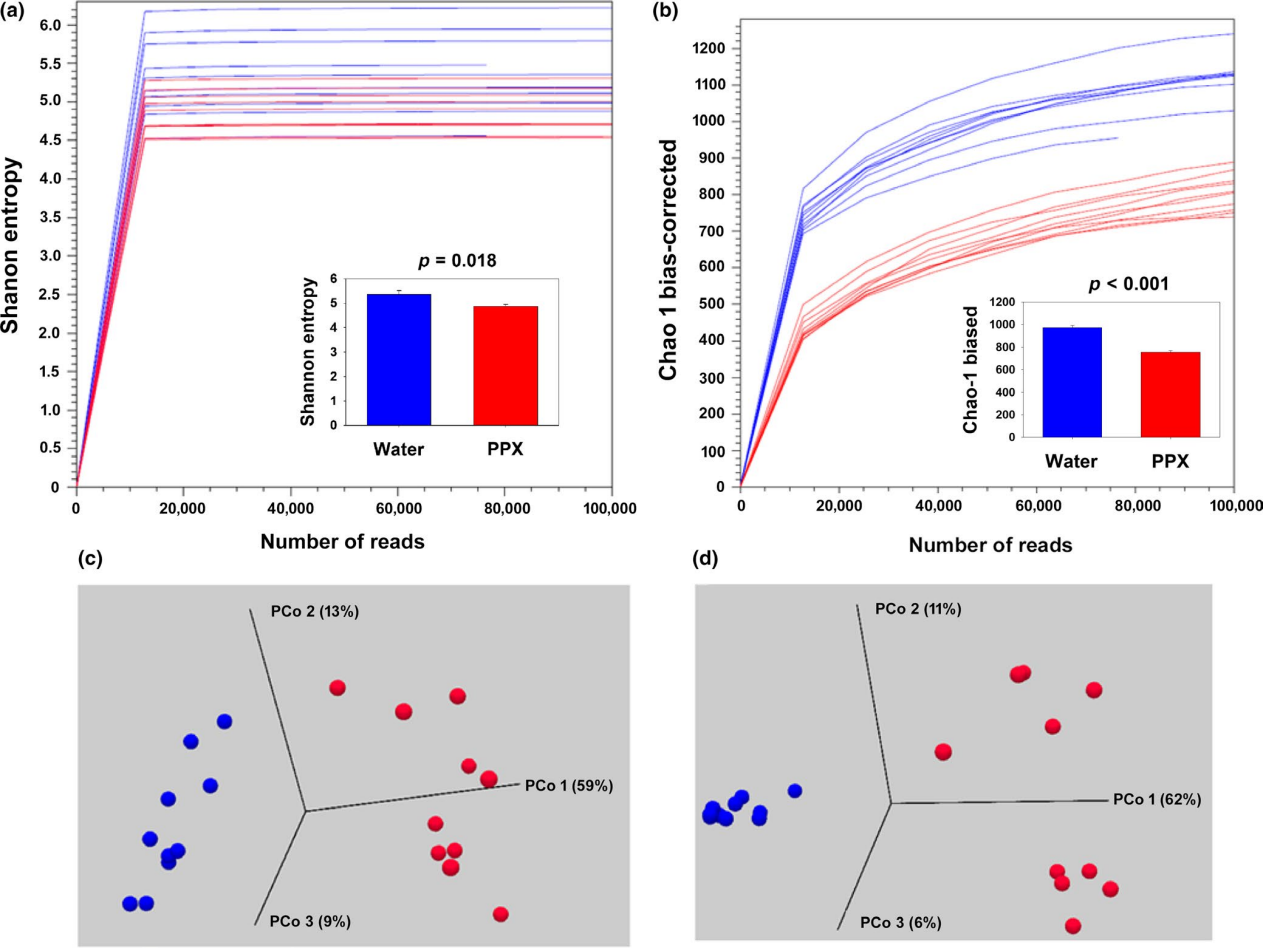
Alpha‐ and beta‐diversity plots for water‐ or PPX‐treated mice. Mice were orally gavaged with water or PPX for 14 days and fecal pellets collected for 16S rRNA gene analysis. Within sample, phylogenetic diversity was quantified using measures of alpha diversity, (a) Shannon entropy, and (b) Chao‐1 bias‐corrected plots. The mean diversity values are plotted in the inset, and there is a significant reduction in diversity in the PPX samples compared water. The bar graphs show the mean ± SE of values obtained with samples rarefied to 73,684 reads. Beta diversity measured using the (c) weighted UniFrac or (d) Bray–Curtis dissimilarity plot showed distinct separation of water and PPX samples with PCo1 accounting for 59% and 62%, and PCo2 13 and 11% of the diversity, respectively. Blue lines, spheres, and bars—water‐treated samples; Red lines, spheres, and bars—PPX‐treated samples

To determine whether treatment with PPX altered the microbiome, mice were treated with either PPX or water for 14 days and their fecal pellets collected for subsequent 16S rRNA gene sequencing analysis. By a variety of metrics, there was a significant less diversity in the microbiome of PPX‐treated mice as illustrated by the plots showing the Shannon entropy of each probability vector (Figure 1a), Chao 1 bias‐corrected index (Figure 1b), and the phylogenetic diversity (Figure S1). PPX treatment resulted in significant changes to the beta diversity (between samples) of the microbial community when compared to water‐treated mice (weighted UniFrac distances, Figure 1c, and Bray–Curtis dissimilarity plot, Figure 1d, *p* < 0.001). Interestingly, the Bray–Curtis dissimilarity plot shows a tight clustering of samples from water‐treated mice but two distinct clusters for samples from PPX‐treated mice that segregated based on cage of origin. No such difference was noted in the mice prior to the onset of treatment (data not shown), suggesting two slightly different microbiomes evolved in response to PPX treatment and this will be discussed further below.

**Figure 2:**
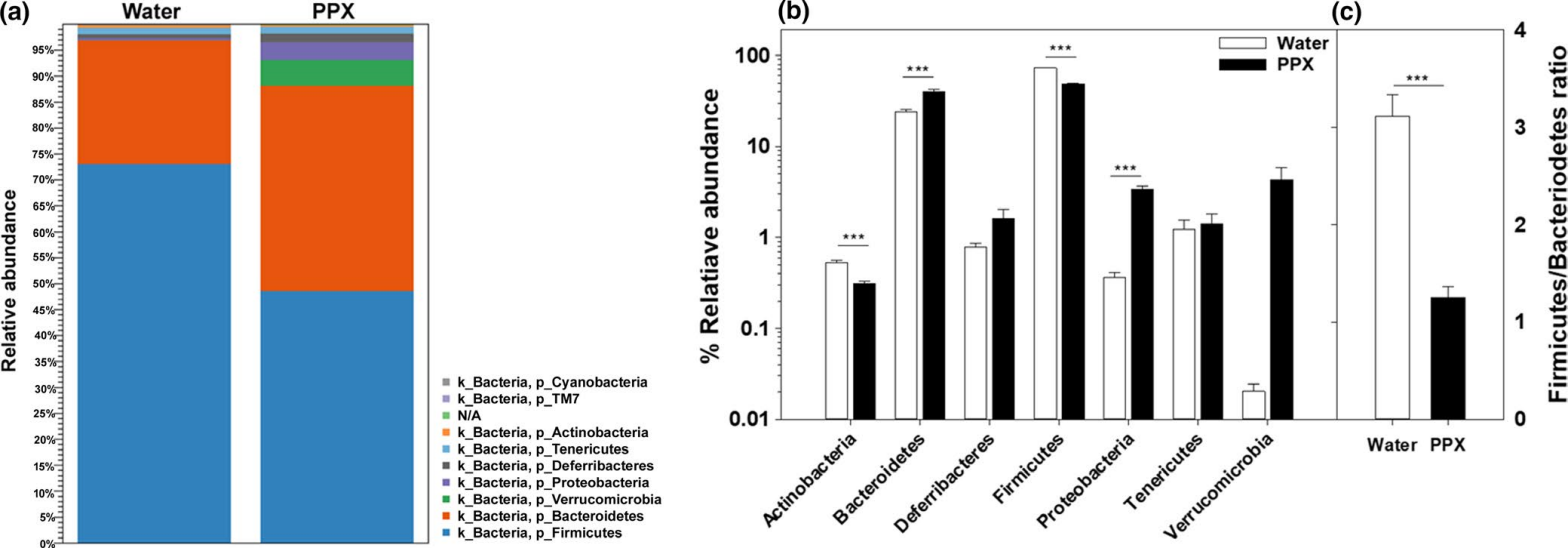
Relative abundance of bacterial phyla in water‐ and PPX‐treated mice. Fecal samples were analyzed for the relative abundance of various phyla, and the results are shown as a stackplot in panel a. A bar plot showing statistical significance of different phyla is shown in panel b, and panel c shows the decrease in the Firmicutes/Bacteroidetes ratio in PPX‐treated mice. ***indicates *p* < 0.001

The relative abundance of OTUs assigned to various bacterial phyla is shown in Figure 2. More than 85% of the OTUs were identified as Bacteroidetes and Firmicutes regardless of treatment (Figure 2a,2). However, the Firmicutes/Bacteroidetes ratio differed depending on treatment. A significantly decrease in the Firmicutes/Bacteroidetes ratio was found in samples from PPX‐treated mice (Figure 2c; *p* < 0.001) resulting from a decrease in Firmicutes and an increase in Bacteroidetes. Members of several other phyla present at low levels also changed. Actinobacteria decreased while members of Proteobacteria increased in fecal samples of PPX‐treated mice (Figure 2a,2; *p* < 0.001). Of note, within the PPX‐treated samples, mice from one of the two cages of had higher levels of the class Verrucomicrobiae, which continued down to the species level with *A. muciniphila* being elevated in one cage while the other cage had very low levels as observed in samples from water‐treated mice. The same was true for the genus *Coprobacillus*, while the genera *Blauta* and *Rosburia* were elevated in samples from both PPX‐treated cages but was greater in the cages with elevated *A. muciniphila*. These cage differences are the likely source of the segregation of the two cages of PPX‐treated mice observed in the Bray–Curtis dissimilarity plot.

**Figure 3:**
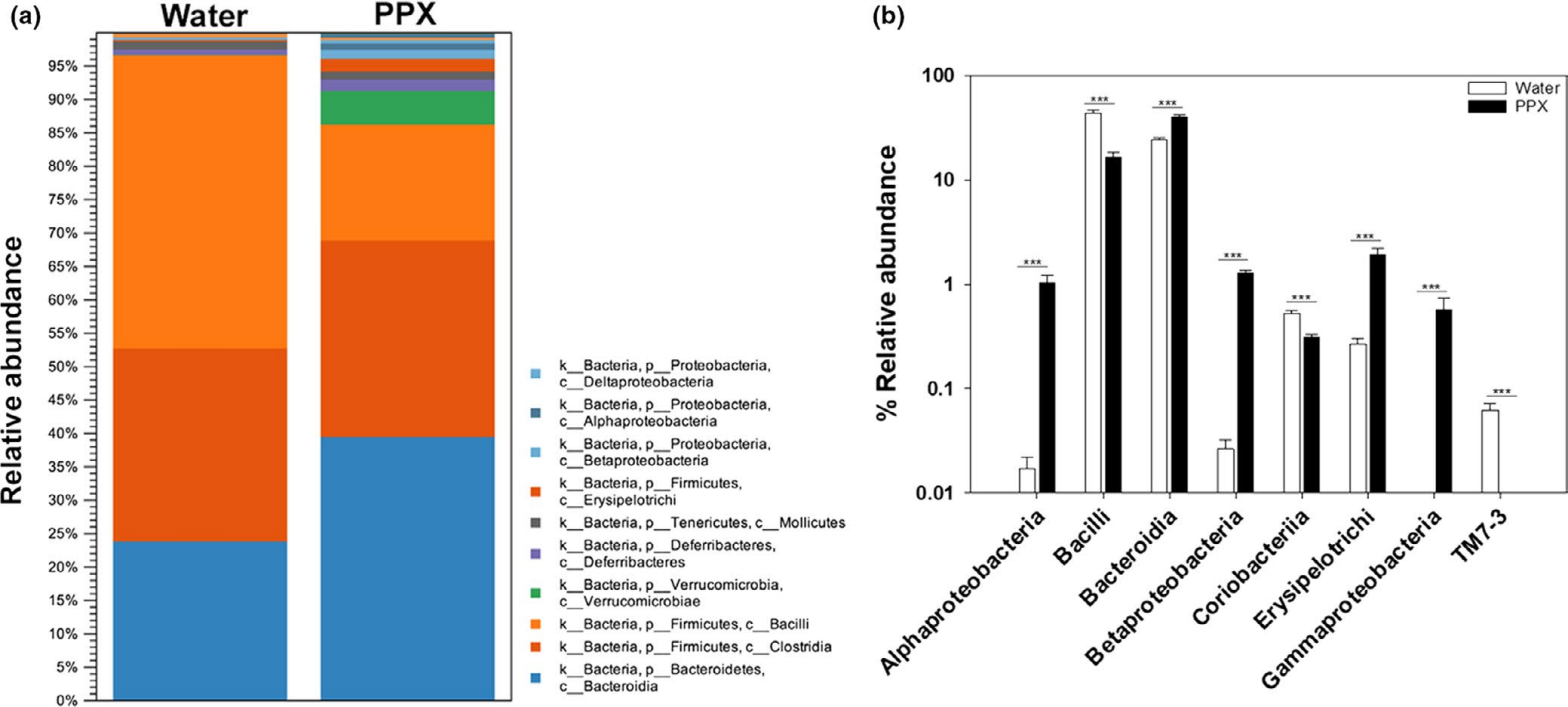
Relative abundance of bacterial classes in water‐ and PPX‐treated mice. Fecal samples were analyzed for the relative abundance of various classes, and the results are shown as a stackplot in panel a. A bar plot showing statistical significance of different classes is shown in panel b. ***indicates *p* < 0.001

The overall percent abundance and microbiota diversity at the class level are shown in Figure 3. Bacilli, Bacteroidia, and Clostridia comprised most of the bacteria from both water‐ and PPX‐treated fecal samples. As was seen at the phylum level with Bacteroidetes, Bacteroidia were elevated by PPX treatment. In contrast, bacilli (essentially all Lactobacillales) were substantially reduced while Clostridia showed similar abundance in water‐ and PPX‐treated fecal samples. Alpha‐, Beta‐, and Gammaproteobacteria were increased by PPX treatment, going from very low levels (<0.05%) in samples from water‐treated mice to the 0.5%–1% range in samples from PPX‐treated mice. Members of the class Erysipelotrichia also increased in response to PPX treatment.

**Figure 4:**
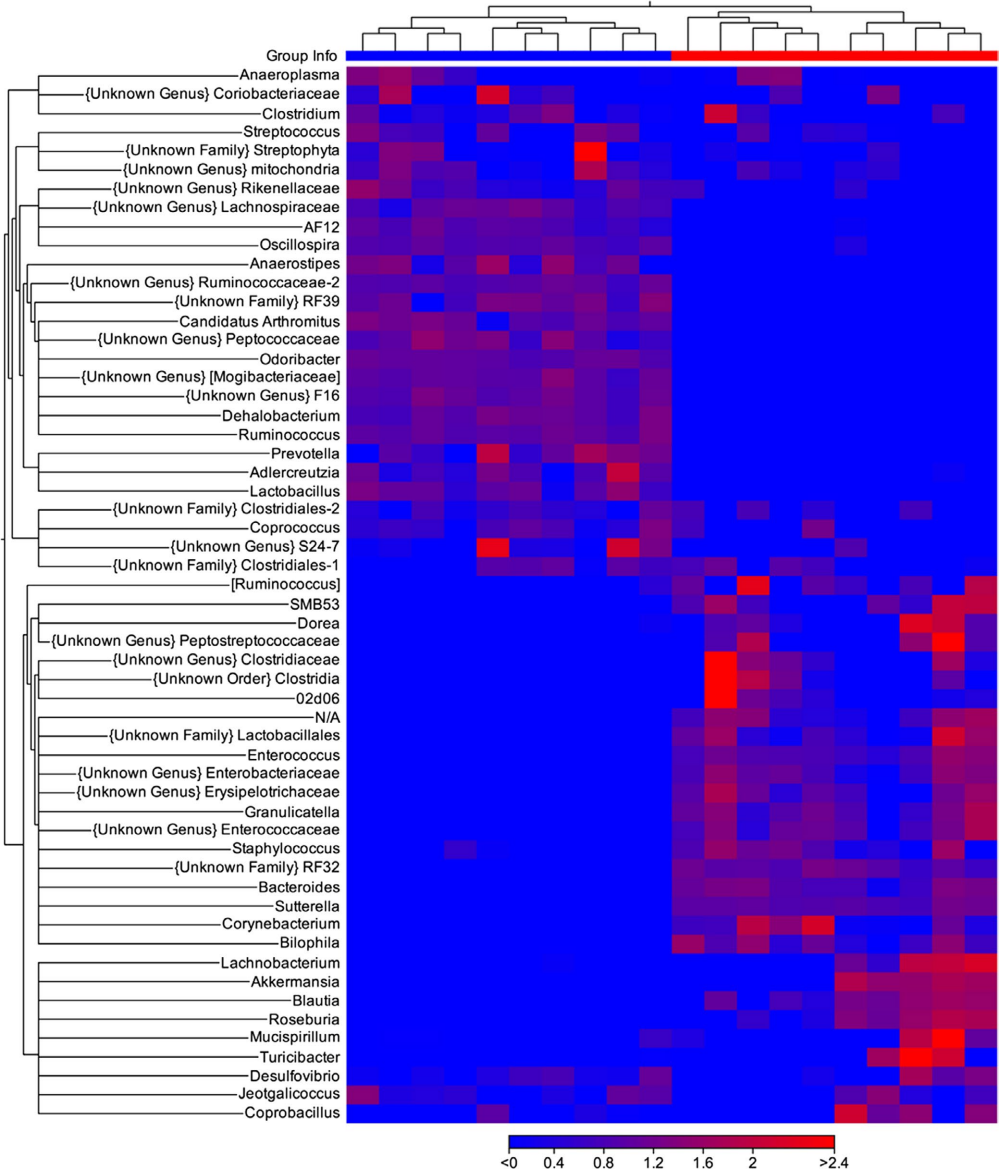
Heatmap showing the relative abundance of bacterial genera in water‐ and PPX‐treated mice. Euclidean heatmaps were generated and show a unique pattern of abundance for various genera in water‐ (top blue bar) or PPX‐treated (top red bar) mice. Note that for the PPX samples there is a set of genera that elevated in one but not the other cage

The relative abundance at the order, family (Figure S2), and genus levels is shown as heatmap correlation plots (Figure 4), and differences are summarized in Table S1. As can be seen in the maps, there are distinct patterns of abundance that differentiate samples from the water and PPX groups, indicating that PPX treatment leads to profound changes to the microbiome that included at the order level an increase in abundance of Burkholderiales, Erysipelotrichales, RF‐32, Enterobacteriales, and Verrucomicrobiales, and a decrease in abundance of CW040, Lactobacillales, and RF39. At the family level, *Alcaligenaceae*, *Bacteroidaceae*, *Erysipelotrichaceae,* and *Verrucomicrobiaceae* were increased while *Lactobacillacieae* and *Ruminococcaceae* were decreased with these patterns persisting at the genus level (Figure 4; Table S1).

### 3.2 Effect of PPX treatment on the microbiome of *Cr*‐infected mice

**Figure 5:**
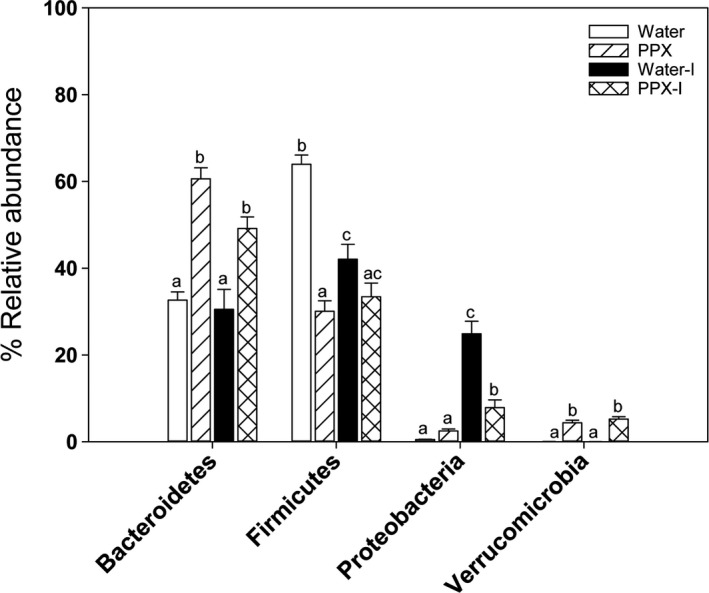
Comparison of the relative abundance of phylum in uninfected and infected water‐ and PPX‐treated mice. Fecal samples were collected prior to and at day six postinfection for analysis. Bacteroidetes levels were affected by treatment but not by infection whereas Firmicutes decreased in water‐treated mice in response to infection. There was a large bloom of Proteobacteria in water‐treated mice that was significantly reduced by PPX treatment

**Figure 6:**
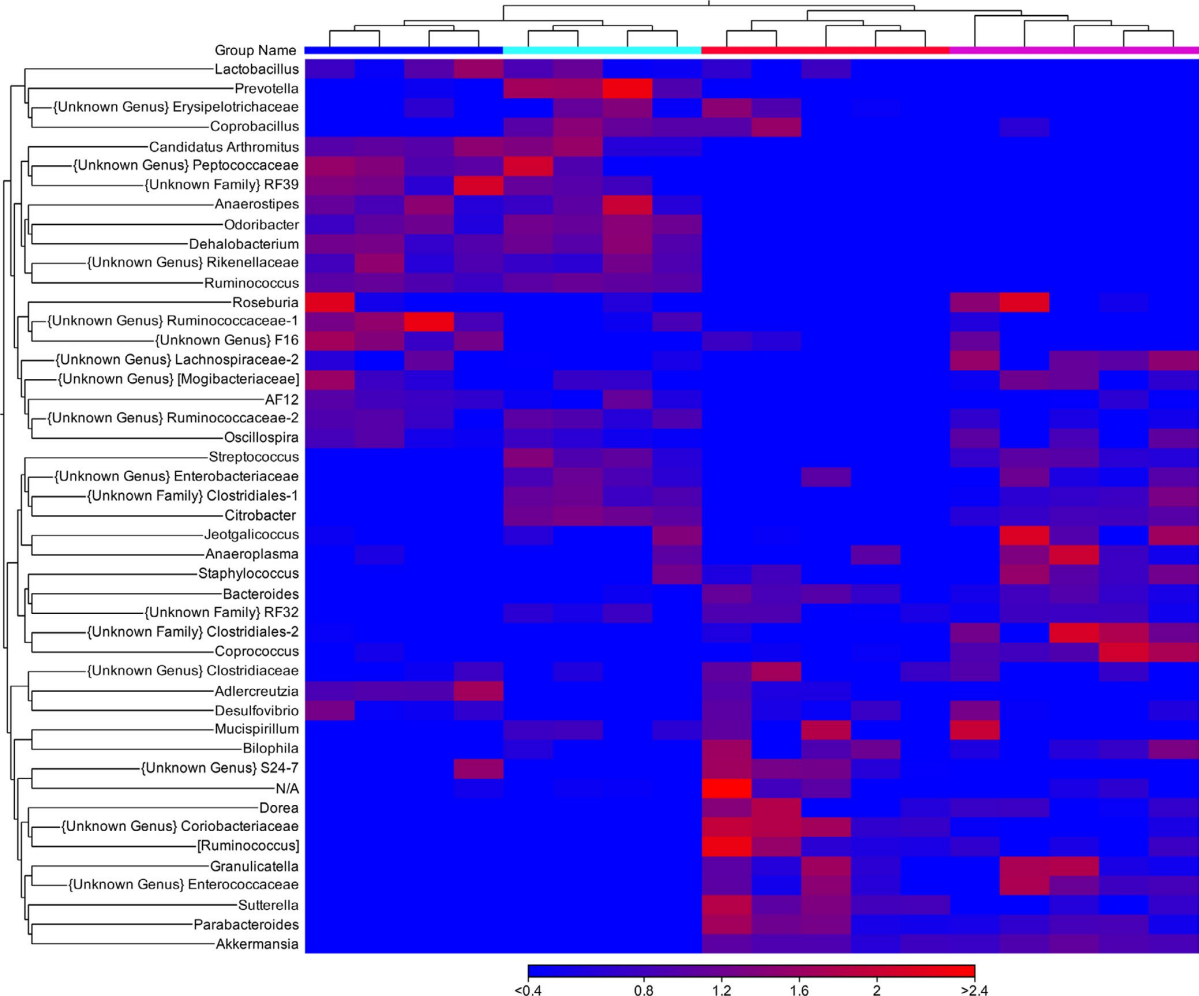
Heatmap showing the relative abundance of bacterial genera in infected and uninfected water‐ and PPX‐treated mice. Euclidean heatmaps were generated and show a unique pattern of abundance for various genera in water‐ (top blue bar) or PPX‐treated (top red bar) mice. Infection shifted the pattern of abundance more in PPX‐treated mice. Water‐infected—cyan bar, PPX‐infected—magenta bar

In the second experiment, mice were treated with PPX or water for 14 days, fecal samples were collected, and then, the mice were infected with *Cr*. At day six postinfection, when peak colonization has been achieved, additional fecal samples were also collected for analysis. As observed in experiment 1, the overall diversity of samples from uninfected PPX‐treated mice was lower than the diversity observed in samples from uninfected water‐treated mice. However, no significant differences in diversity were detected in samples from *Cr*‐infected mice treated with water (water‐I) or PPX (PPX‐I) (Figure S3a). In the beta‐diversity plots (Figure S3b), a clear separation of the four groups is shown (*p* < 0.001). The relative abundance of select phyla is shown in Figure 5. While PPX treatment increased the relative abundance of Bacteroidetes and Verrucomicrobia, levels essentially remained unchanged after infection in both water‐I and PPX‐I mice while Firmicutes decreased in water‐I but not PPX‐I mice. Although Proteobacteria were present at low levels in uninfected mice, a significant bloom occurred after infection, increasing in both water‐I and PPX‐I mice; however, the increase was 3‐fold higher in water‐I mice reaching 25% relative abundance. In general, samples segregated by treatment and infection in relative abundance heatmaps for orders, families (Figure S4), and genera (Figure 6). Table S2 shows the relative percent abundance of various genera. Several minor genera, including *RF39, Peptococcaceae, Anaerostripes, Dehalobacterium*, and *Candidatus Arthromitus*, were essentially absent in feces from PPX‐I mice but present in water‐I mice. While infected and uninfected mice had similar abundance of certain families and genera, there were also families and genera with altered abundance resulting from infection that was more pronounced in water‐treated mice. As was observed in uninfected mice, *Bacteroides* was lower in water‐I than PPX‐I mice but increased in water‐I mice from low levels in uninfected mice while it remained unchanged in PPX‐I mice. As previously reported (Hoffmann et al., 2009), *Lactobacillus* levels decreased significantly in response to infection in both groups, but as a percentage was higher in water‐I than PPX‐I mice. Also, *Coprococcus, Desulfovibrio, and Parabacteriodes* were lower in water‐I than PPX‐I mice while *Sutterella* and *Provetella* increased in response to infection in water‐I but not PPX‐I mice.

**Figure 7:**
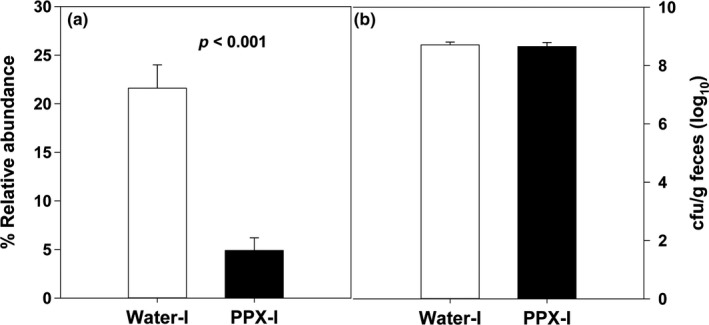
The effect of infection on the relative abundance of Citrobacter at the genus level and the level of fecal excretion of Cr in water‐ and PPX‐treated mice. The relative abundance of the genus Citrobacter is significantly higher in water‐ versus PPX‐treated mice (panel a). The level of Cr excretion on day 7 postinfection in feces is shown in panel b

Of the four classes comprising the phylum Proteobacteria that were found in the fecal samples, the increase in infected samples was mainly due to Gammaproteobacteria (to which *Cr* belongs). Bacilli levels decreased in both water‐I and PPX‐I mice but remained higher in water‐I than PPX‐I mice. Similarly, the relative abundance of the order Enterobacteriales, and family, *Enterobacteriaceae* (to which *Cr* belongs) was 25%. Twenty‐two and five percent of the sequences mapped to the genus *Trabulsiella*, species, *farmeria*, in water‐I and PPX‐I mice, respectively. However, while the Greengenes lineage is *Proteobacteria; Gammaproteobacteria; Enterobacteriales; Enterobacteriaceae; Trabulsiella;* and *farmeria,* the European Nucleotide Archive indicates the Greengenes lineage is incorrect with the correct linage being *Proteobacteria; Gammaproteobacteria; Enterobacteriales; Enterobacteriaceae; Citrobacter; and farmeria,* indicating that the increase is due to *Citrobacter* and not *Trabulsiella*. Furthermore, the higher abundance of *Cr* in the samples from water‐I versus PPX‐I mice was confirmed by shotgun sequencing with specific mapping to *Cr* (data not shown). The difference in relative abundance between water‐I and PPX‐I mice (Figure 7a) contrasts with the nearly identical fecal levels of viable *Cr* obtained from plating fecal homogenates on selective media (Figure 7b). Similar results are obtained from homogenized colon tissue (data not shown). These results indicate the relative abundance of *Cr* rather than the absolute number of *Cr* is greater in a water‐I versus PPX‐I mice.

## 4 DISCUSSION

Polyphenols are micronutrients ubiquitously present in many fruits and vegetables that are thought to have health promoting properties. Pomegranate has been heavily promoted as a “super” food mostly due to its high polyphenol content. Pomegranate juice or peel extracts have been shown to provide protection against chemical‐induced colitis (Kim et al., 2016; Larrosa et al., 2010; Rosillo et al., 2012). Pomegranate peel possesses higher concentration of polyphenols than the edible portion, and it reduced *Cr*‐induced colonic damage in PPX‐treated mice (Smith *et al*., manuscript submitted). Here, we demonstrate that oral administration of a PPX can significantly alter the host microbiome of the *Cr*‐susceptible mouse strain, C3H/HeNCr, and reduce overgrowth of *Cr*.

Of significance, PPX treatment decreased the extent of the *Cr* bloom after infection from about 22% relative abundance in mice receiving water to 5% in mice treated with PPX. In contrast, the number of viable *Cr*/g of feces or tissue (data not shown) was the same suggesting similar absolute levels of *Cr* in the colon. The high relative abundance of *Cr* reported here is much higher than reported for C57Bl/6 mice (<1% at day 9 postinfection; (Hoffmann et al., 2009)). Infection of mice with *Cr*, but not *Citrobacter jejuni*, a bacterium that can colonize the mouse colon but does not induce pathology, caused a marked reduction in total intestinal microbes (Lupp et al., 2007), thus effectively raising the relative abundance of *Cr*. The fourfold increase in *Cr* abundance in water‐I mice over PPX‐I‐treated mice suggests that PPX treatment made the microbiome more resistant to displacement by the subsequent infection with *Cr*, thus reducing the percentage of the total microbiome that *Cr* occupies rather than reducing the absolute number of *Cr* and correlates with the increased colon pathology observed in water‐treated versus PPX‐treated mice.

Alterations and/or reduction in the number of commensals may be deleterious since commensal bacteria have been shown to be important for control of *Cr* infections (Kamada, Chen, Inohara, & Nunez, 2013; Kamada et al., 2012; Lamas et al., 2018). The microbiota plays a significant role in the infection process, and studies indicate that it is possible to manipulate the microbiome through diet (Desai et al., 2016; Kamada et al., 2012), and probiotics (Collins, Chervaux, et al., 2014; Vong et al., 2015) to minimize the extent/severity of *Cr* infections. Furthermore, susceptibility to *Cr* is mouse strain dependent and fecal transplants between resistant and susceptible strains can alter susceptibility (Ghosh et al., 2011; Willing et al., 2011). Notably, protection of mice from lethal colitis was associated with increased levels of *Bacteroidetes* (Ghosh et al., 2011) and we previously observed decreased mortality and pathology in PPX‐treated mice (Smith *et al*., manuscript submitted).

Here, we found that treatment with PPX significantly increased the presence of Bacteroidetes in both uninfected and infected mice while decreasing Firmicutes thus lowering the Firmicutes/Bacteroidetes ratio. Increased levels of *Bacteroides* are generally considered beneficial. Monocolonization of germ‐free mice with *Bacteroides fragilis* protected against DSS‐induced acute colitis (Chiu, Ching, Wang, & Liu, 2014). *Bacteroides* also can provide a pool of short‐chain fatty acids that can be utilized by the host and other metabolic substrates that can be used by other commensals (Wexler, 2007). In addition, Bacteroidales were shown to be sufficient to promote intraepithelial cell presence and cytokine secretion in the colon, thus promoting epithelial barrier function in response to *Cr* infection (Kuhn et al., 2017). Thus, at least part of the protective effects derived from PPX consumption is likely driven by the increase in *Bacteroides*.

Diet can have significant effects on the microbiome. Feeding mice a high‐fat diet led to a decrease in Bacteroidetes (Hildebrandt et al., 2009) and increases the Firmicutes/Bacteroidetes ratio (Turnbaugh, Backhed, Fulton, & Gordon, 2008). In addition, the ratio was increased in obese individuals and decreased with weight loss (Ley, Turnbaugh, Klein, & Gordon, 2006; Turnbaugh et al., 2008). A cranberry extract, however, was unable to reverse the high‐fat, high‐sugar (HF/HS)‐induced decline of Bacteroidetes (Anhe et al., 2015). In contrast, a decrease in the Firmicute/Bacteroidetes ratio was observed in mice fed a high‐fat diet supplemented with oolong, green, or black tea polyphenols (Cheng et al., 2018; Henning, Yang, et al., 2017) or grape polyphenols (Roopchand, Carmody, Kuhn, & Moskal, 2015). Mice fed a HF/HS diet alone or in conjunction with a commercial extract of pomegranate by‐product (POMx, POM Wonderful) had very low levels (<0.01%) of the genus *Bacteroides* that dropped to undetectable levels upon POMx treatment and high relative abundance of the family S24‐7 (58%), a member of the Bacteroidetes that was not altered by POMx treatment (Zhang et al., 2017). We also saw no effect of PPX treatment on the relative abundance of the family S24‐7 but saw less of this family (approximately 16%) in both water‐ and PPX‐treated mice and more *Bacteroides* that was significantly increased by PPX treatment.

Polyphenol consumption can significantly alter the microbiome. Pomegranate ellagitannins were shown to reduce starch digestibility in vitro (Bellesia, Verzelloni, & Tagliazucchi, 2015) and may alter the amount of starch available for fermentation in the cecum and colon which can alter the microbiome and production of short‐chain fatty acids (Wong, Souza, Kendall, Emam, & Jenkins, 2006). Feeding tea polyphenols increased the abundance of *Parabacteroides, Bacteroides*, and *Prevotella*, while significantly decreasing several genera such as *Roseburia, Lactobacillus, Blautia, Anaerostipes, Shuttleworthia, Bryantella*, and *Lactococcus* in the context of a HF/HS diet in diet‐induced obese mice (Henning, Yang, et al., 2017). *A. muciniphilia* has been associated with improved health outcomes, especially metabolic disorders (Derrien, Belzer, & Vos, 2017; Schneeberger et al., 2015). Dietary polyphenols have been reported to increase the prevalence of *A. muciniphilia* (Anhe et al., 2015; Roopchand et al., 2015), and in humans, this was associated with the ability to metabolize pomegranate ellagitannins to urolithin A (Henning, Summanen, et al., 2017; Li, Henning, et al., 2015). However, POMx treatment of mice did not increase the relative abundance of *A. muciniphilia* while treatment with inulin did (Zhang et al., 2017). The same study showed that the *Turicibacteraceae* and *Rumminococcaceae* were increased by POMx treatment of mice fed a HF/HS diet. We saw an increase in *A. muciniphilia* in some but not all PPX‐treated mice; thus, it is unlikely that it is associated with decreased *Cr*‐induced pathology.

The relative abundance of *Lactobacillus* was surprisingly high in our experiments but dropped dramatically in response to PPX treatment. Polyphenols have been reported to positively or negatively affect the growth of some strains of *Lactobacilli *in vitro that was compound and species specific (Duda‐Chodak, 2012; Tabasco et al., 2011). Red wine polyphenols increased *Lactobacilli* levels in obese human subjects (Moreno‐Indias et al., 2016). As we observed with our extract, POMx enhanced the growth of Proteobacteria, as well as total bacteria, *Bifidobacterium* spp., and *Lactobacillus* spp. from human feces (Bialonska et al., 2010; Li, Summanen, Komoriya, & Henning, 2015) but inhibited the growth of *B. fragilis*, Clostridia, and *Enterobaceriaceae* (Li, Summanen, et al., 2015). Crude pomegranate juice and peel extracts inhibited in vitro growth of pathogenic clinical isolates of *E. coli* and *Staphylococcus aureus* and other pathogenic bacteria (Howell & D'Souza, 2013; Pagliarulo et al., 2016) and swarming activity of *Cr* (John et al., 2017). However, fecal excretion and colonic tissue burden of *Cr* were not affected by PPX treatment (Figure 7b and Smith *et al.,* submitted manuscript), indicating that PPX treatment was not inhibiting growth of *Cr*. While some *Lactobacillus* species including *L. acidophilus* and *L. casei* are considered probiotics, other species of *Lactobacillus* (e.g., *L. reuteri*) have been associated with obesity (Million et al., 2012). In patients with ulcerative colitis, seven *Lactobacillus* strains, including *L. acidophilus,* and *L. casei,* were decreased while *L. crispatus, L. delbrueckii,* and *L. reuteri* increased (Cui et al., 2016). Furthermore, mice treated daily with *L. crispatus* had more severe DSS‐induced colitis whereas *L. plantarum* treatment improved DSS‐induced colitis (Cui et al., 2016), suggesting that beneficial effects of *Lactobacillus* are species dependent and that decreased prevalence of *Lactobacilli* may not inherently be deleterious.

Somewhat surprising was the decrease in diversity observed in samples from PPX‐treated mice compared to those from water‐treated mice. Generally, increased diversity is considered to promote a healthy microbiome and dysbiosis is associated with decreased diversity (Levy, Kolodziejczyk, Thaiss, & Elinav, 2017). No difference in diversity between the two treatments was apparent after infection. PPX treatment did, however, result in a significantly altered microbiome as observed in the β‐diversity plots and the abundance heatmaps from both uninfected and infected PPX‐treated mice. This suggests that the microbiome induced by PPX is more resistant to displacement by *Cr* thus keeping the relative abundance of *Cr* lower resulting in less colonic pathology. Also, surprising was the decrease in abundance of *Candidatus Arthromitus* in PPX‐treated mice. This bacterium was originally thought to only be associated with terrestrial arthropods but recent reports indicate that that it is a segmented filamentous bacterium (family Lachnospiraceae) (Bolotin et al., 2014; Thompson, Vier, Mikaelyan, Wienemann, & Brune, 2012) with immunomodulating properties (Gaboriau‐Routhiau et al., 2009; Ivanov et al., 2009, 2008). Segmented filamentous bacteria are important for the development of Th17 immunity and resistance to *Cr* (Ivanov et al., 2009) but its reduction by PPX treatment had no apparent effect.

Gastrointestinal infections can induce dysbiosis, and the effect of *Cr* on the microbiome of C57Bl/6 mice fed standard chow has been investigated (Collins, Chervaux, et al., 2014; Hoffmann et al., 2009). Hoffman et al. (2009) found that the microbiome was more affected late after infection (day 14) than at day 9. In agreement with their findings, in water‐I mice we saw reduced abundance of *Lactobacillaceae* after infection and an outgrowth of some Proteobacteria including Alphaproteobacteria and Gammaproteobacteria while Deltaproteobacteria decreased. In our case, the large increase in Gammaproteobacteria is primarily due to *Cr*, a member of this class and was much higher than reported by Hoffmann et al. (2009). C3H/HeN mice are more susceptible to *Cr* infection than C57Bl/6 mice (Vallance et al., 2003), and this may be related to the higher relative abundance of *Cr* after infection in C3H/HeN mice. The fact that we did not see all the same changes reported by Hoffman et al. (2009) may result from different mouse strains (with different susceptibilities to *Cr* infection) and sampling times as they showed distinct time‐dependent changes. Collins, Chervaux, et al. (2014), Collins, Keeney, et al., 2014 reported that *Rumminococcus* decreased in response to *Cr* infection and loss of *Rumminococcus* is associated with susceptibility to DSS‐induced colitis (Zenewicz et al., 2013). PPX, but not water treatment, resulted in a dramatic decline in *Rumminococcus* levels but this was not associated with increased *Cr*‐induced pathology.

In conclusion, PPX treatment dramatically alters the microbiome of mice. PPX treatment decreases the Firmicutes/Bacteroidetes ratio primarily by raising Bacteroidetes levels. Several minor bacterial species also are altered in response to PPX treatment resulting in a modified microbiome. The PPX‐induced microbiome is more resistant to overgrowth by *Cr* infection resulting in significantly lower relative abundance of *Cr* even though the number of viable *Cr*/g of feces is the same. Our results indicate that pomegranate peels, an agricultural waste product, may be a source of compounds that are beneficial for treatment of intestinal disorders but further research is required.

## CONFLICT OF INTEREST

The authors have no conflicts of interest to declare.

## ETHICAL REVIEW

The animal studies described in this manuscript were approved by the USDA/ARS Beltsville Institutional Animal Care and Use Committee.
