# Supplementary material for: Pomegranate peel extract alters the microbiome in mice and dysbiosis caused by Citrobacter rodentium infection
Source: Food Sci Nutr. 2019 Jul 7;7(8):2565–76. doi: 10.1002/fsn3.1106 (PMC6694437; doi:10.1002/fsn3.1106)
Supplement: Supplementary file 1 [file FSN3-7-2565-s001.docx]

**Supplemental Figures.**

**Figure S1.**


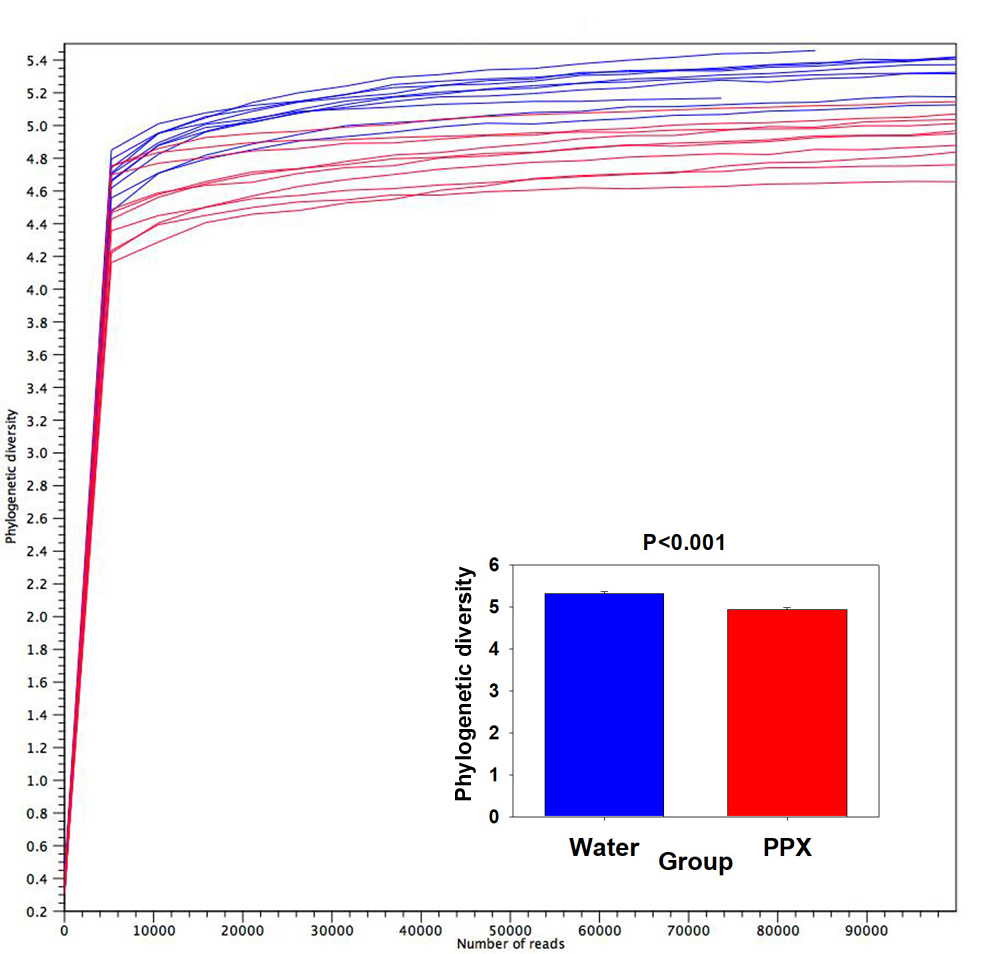


**Figure S1. Phylogenetic diversity plot for water and PPX-treated mice.** Mice were orally gavaged with water or PPX for 14 days and fecal pellets collected for 16S rRNA gene analysis. The mean diversity values are plotted in the inset and there is a significant reduction in diversity in the PPX samples compared water. Blue lines and bars – water-treated samples; Red lines and bars – PPX-treated samples.

**Figure S2.**


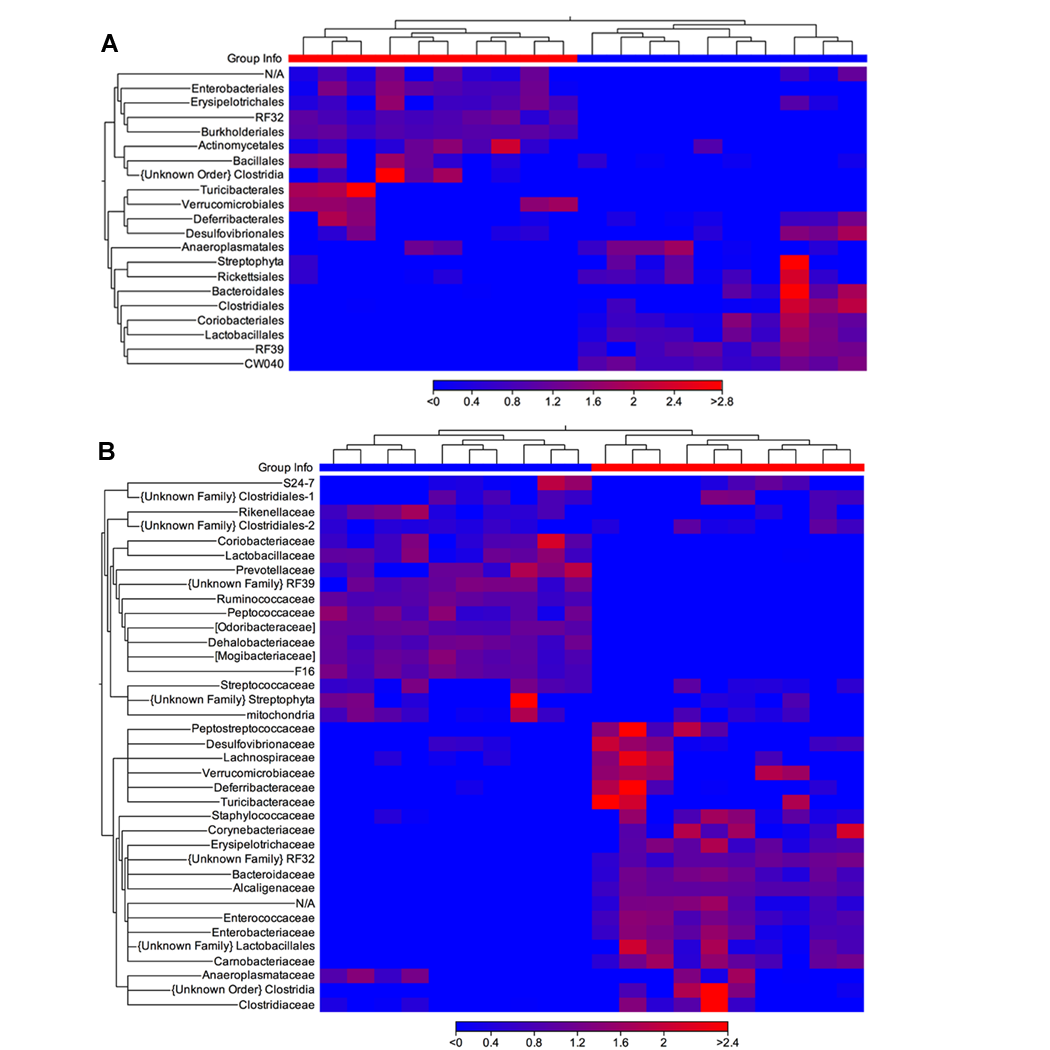


**Figure S2. Heat map showing the relative abundance of bacterial orders and families in water and PPX treated mice.** Euclidean heatmaps were generated and show a unique pattern of abundance for various orders (A) and families (B) in water- (top blue bar) or PPX-treated (top red bar) mice. Note that for the PPX samples there is a set of families that elevated in one but not the other cage.

**
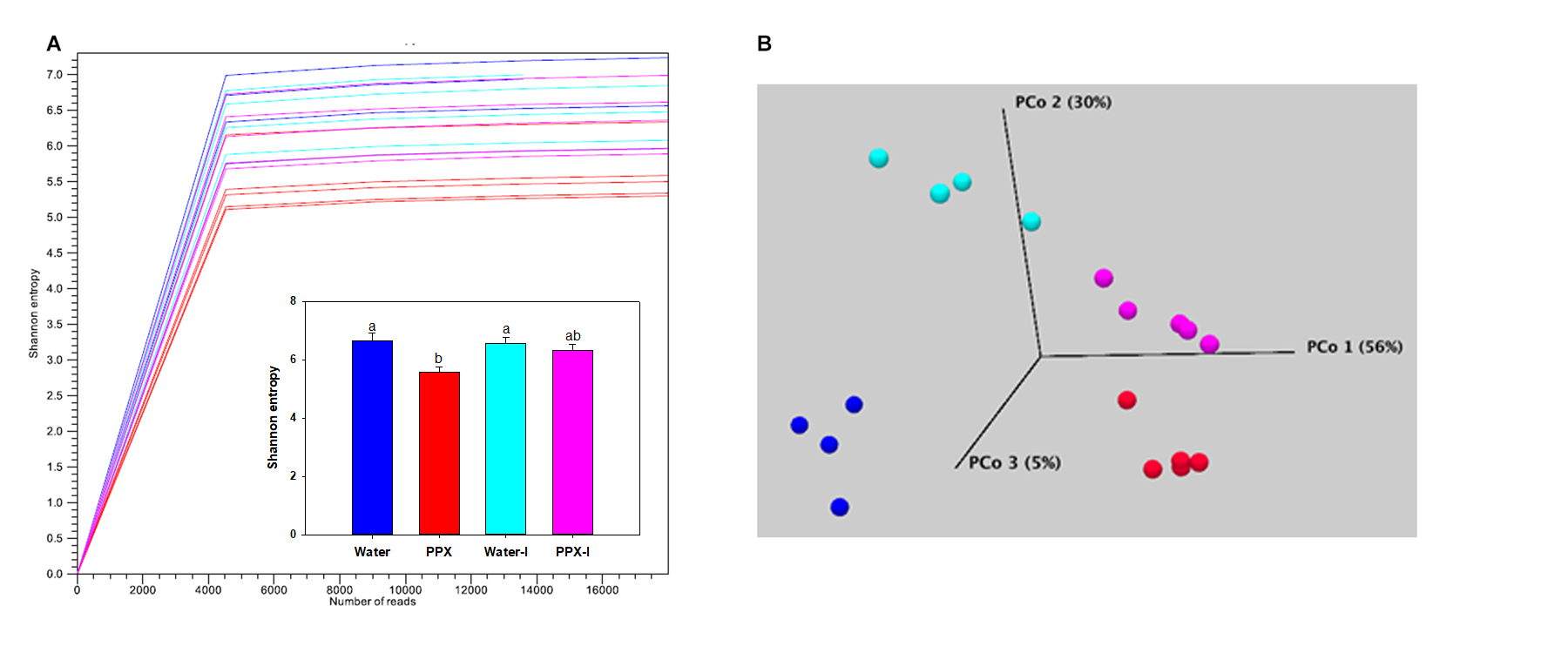
Figure S3.**

**Figure S3. Alpha- and Beta-diversity plots for water and PPX-treated mice before and after infection.** Mice were orally gavaged with water or PPX for 14 days and fecal pellets collected for 16S rRNA gene analysis. A set of mice was then infected with *Cr* and fecal samples collected at day 6 post-infection for 16S rRNA gene analysis. Panel A) Shannon entropy plot for α-diversity. The mean ± SE diversity values (rarified to 13589) are plotted in the inset and there is a significant reduction in diversity in the uninfected PPX samples compared water that was not present after infection. Panel B) Weighted UniFrac plot of β-diversity for water (blue), water-infected (cyan), PPX (red) and PPX-infected (magenta).

**Figure S4.**


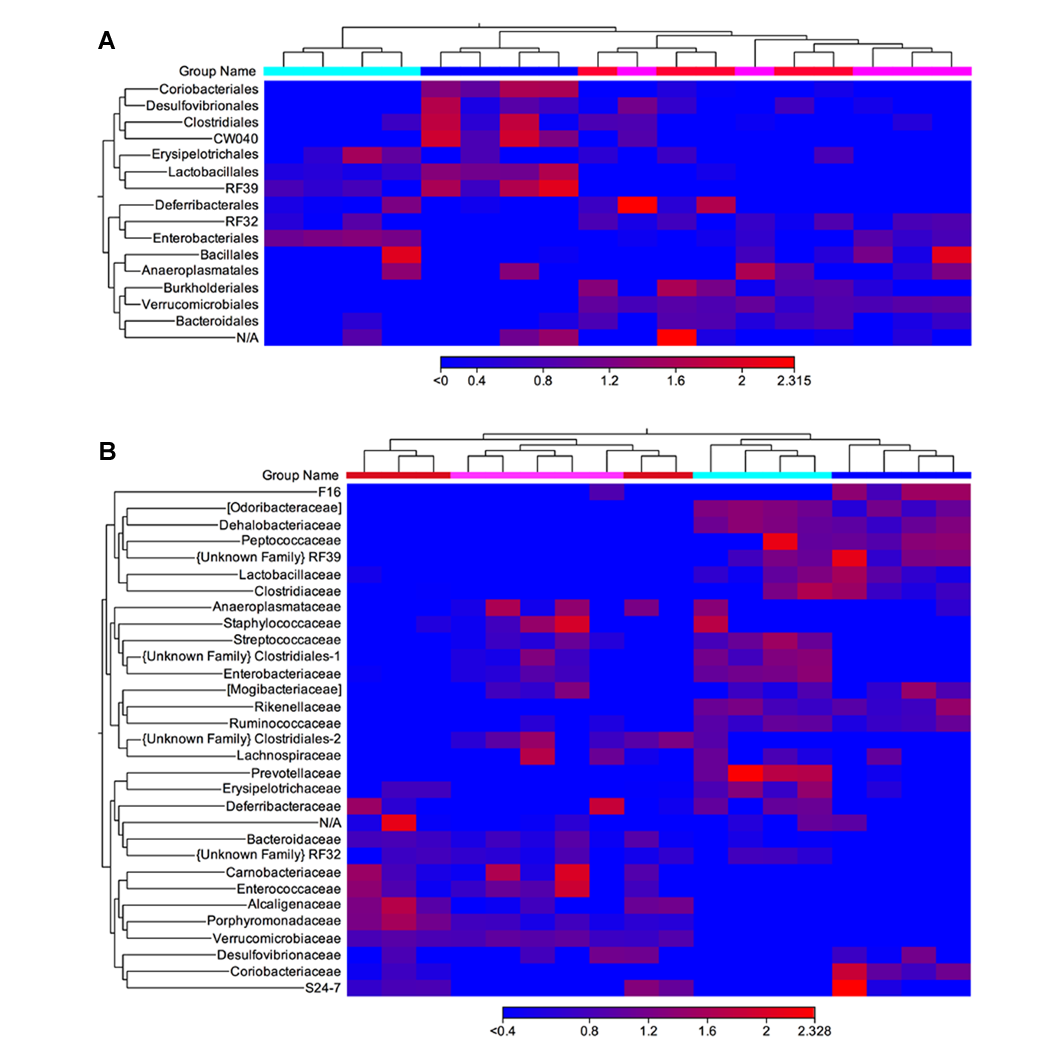


**Figure S4. Heat map showing the relative abundance of bacterial orders and families in infected and uninfected, water and PPX treated mice.** Euclidean heatmaps were generated and show a unique pattern of abundance for various orders (A) and families (B) in water- (top blue bar) or PPX-treated (top red bar) mice. Infection shifted the pattern of abundance both water- and PPX-treated mice. Water infected – cyan bar, PPX infected – magenta bar.
